# Dexamethasone-inducible LhGR/pOp system: simple and flexible spatiotemporal control of gene expression

**DOI:** 10.1007/s00425-026-05123-7

**Published:** 2026-08-17

**Authors:** Zuzanna Majewska, Grzegorz Jursza, Alicja Dolzblasz

**Affiliations:** Department of Plant Developmental Biology, University of Wroclaw, Wroclaw, Poland

**Keywords:** *Arabidopsis*, Dexamethasone, Induction systems, LhGR/pOp, Non-model plants, Resources

## Abstract

**Main Conclusion:**

Our review illustrates how the dexamethasone-inducible LhGR/pOp system has been used across numerous tissues/organs and plant species, and summarizes the resources and inducer application procedures established to date.

**Abstract:**

The establishment of groundbreaking molecular biology tools has enabled the rapid advancement of research based on the model plant *Arabidopsis*, which currently strongly benefits non-model but economically important species. Spatiotemporal control of transgene expression via the chemically inducible system GR-LhG4/pOp has proven to be a particularly powerful, universal and non-invasive experimental approach. This review characterizes the mechanism of action of GR-LhG4/pOp and synthesizes information on available GR-LhG4 transgenic lines and inductor (dexamethasone) application procedures across various plant tissues. Moreover, feasible experimental approaches are depicted that range from classical ones aiming at selected genes overexpression or silencing to highly innovative ones, like those integrating the GR-LhG4/pOp system with CRISPR-Cas for targeted ablation of specific cell types. Information about the existing GR-LhG4/pOp lines from non-*Arabidopsis* plants is also provided, in hope that this sophisticated but universal research tool will become more exploited in crop research.

## Introduction

The fate of each cell within a plant tissue is determined by the spatial and temporal expression of numerous genes, the functions of which, in many cases, have not been fully elucidated. The precise determination of the genetic regulation of various developmental processes was enabled by the widespread use of transgenic *Arabidopsis thaliana* (hereafter referred to as *Arabidopsis*) plants. The obtained knowledge can be more easily translated to other plant species, see e.g. recent focus issue from The Plant Cell “Translational Research from *Arabidopsis* to Crop Plants and Beyond” (e.g. Bevan et al. 2025; Roeder et al. 2025). Phenotyping adult *Arabidopsis* transgenic plants with ubiquitous gene expression or knock-out mutants can be difficult due to pleiotropy and/or accumulation of phenotypic changes over time as the plant ages. To overcome these constraints, different inducible systems providing spatial and/or temporal control over transgene expression are used. These systems enable more accurate analyses of phenotypic changes triggered by the induction or silencing of a particular gene expression (e.g. Craft et al. 2005; Sekeli et al. 2014; Wang et al. 2020; Schubert et al. 2022; Sinha et al. 2023), identification of direct targets of transcription factors (TFs) (Yamaguchi et al. 2015; Shimada et al. 2022), acceleration of plant regeneration from callus cultures (Zhang et al. 2024) or creation of plants overaccumulating medically or agriculturally relevant compounds (Aharoni et al. 2006; Kodaira et al. 2011). Such systems serve as an experimental platform allowing the blocking of specific cellular processes, such as vesicular trafficking (Osterrieder et al. 2010; Adamowski et al. 2018), “labelling” selected cell types for transcriptomic, proteomic, metabolomic profiling (e.g. Černý et al. 2013; Barff et al. 2025) or even the control over cell ablation (Gehrke et al. 2023), all in a spatiotemporally controllable manner.

In chemically inducible systems, a common—but not the only—experimental design, involves a constitutive (e.g. CMV p*35S*) or tissue-specific promoter that drives the expression of a TF, which can then bind to its cognate promoter only in the presence of a chemical inductor. When such binding occurs, the expression of the effector protein of choice will take place. In such chemically inducible systems researchers can trigger transgene expression at desired stages of plant development (via inductor application), and only in specific tissues or cell types (depending on the promoter used). Nevertheless, induction systems also have their limitations. For example, the ethanol-inducible AlcR/AlcA system (based on the AlcR TF and the AlcA promoter from *Aspergillus nidulans*) (Roberts et al. 2005) can, under some circumstances, cause unwanted transgene expression (can be “leaky”), nonspecific growth defects and, in the long term, produce misleading results due to ethanol evaporation and its negative impact on plant tissues (Randall 2021). TFs can be also directly fused to a molecular “tag” to render their activity inducible (i.e. rat glucocorticoid receptor – GR) (Günl et al. 2009). In consequence, the TF-GR fusion protein becomes a conditional switch that triggers the expression of the target genes only in the presence of a glucocorticoid ligand. Notably, various environmentally driven systems, which use promoters induced by a specific abiotic factor (e.g. heat, light) have also been established and are widely used in research (Ochoa-Fernandez et al. 2020; Forestier et al. 2023; Liang et al. 2023). For an overview of the induction systems developed so far for plant research see Zuo et al. (2000), Moore et al. (2006), Borghi (2010), Samalova and Moore (2021), Omelina et al. (2022).

In our review we focus on the very effective, precise and, to the best of our knowledge, non-invasive (not affecting plant growth) GR-LhG4/pOp system. We aim to 1) provide a better understanding of this sophisticated induction system, 2) demonstrate how extensively it has been applied in *Arabidopsis* research so far, by compiling information on generated transgenic lines and DEX application procedures, and 3) offer a perspective on the applicability of the GR-LhG4/pOp system to economically valuable species.

### GR-LhG4/pOp system – spatiotemporal transgene induction in *Arabidopsis* research

#### GR-LhG4/pOp system development

**Fig. 1:**
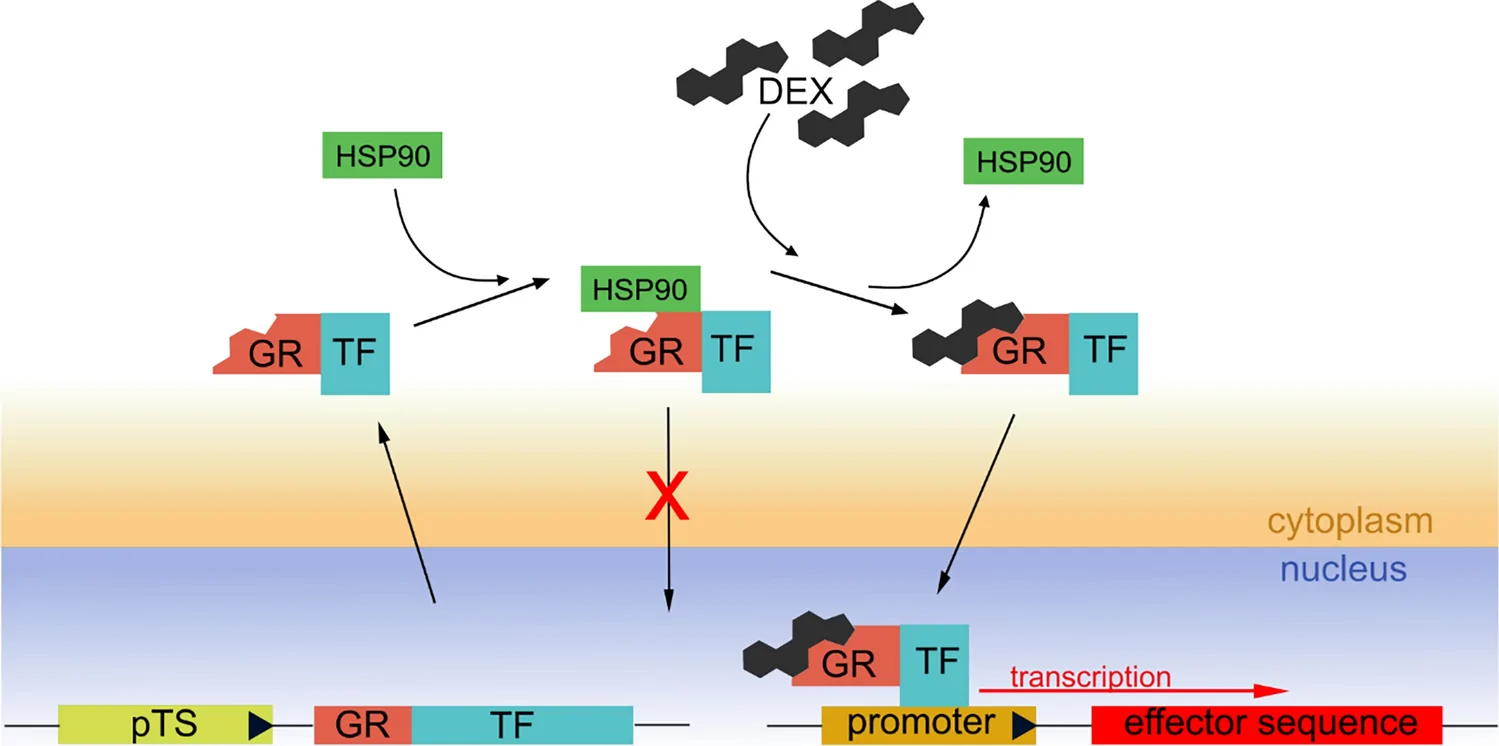
Mode of action of TF-GR lines. In the absence of DEX, GR-fused TF is bound by the HSP90 protein, which prevents TF translocation to the nucleus and thus blocks transcription of its target genes. When DEX is present in the cytoplasm, it binds to GR, which triggers the detachment of HSP90 and enables the TF to translocate to the nucleus, thereby allowing transcription of the target genes (effector sequence shown in red). pTS—tissue specific promoter. Figure prepared using Inkscape version 1.4.2

In the GR-LhG4/pOp system the temporal control over transgene expression is provided by a ligand-binding domain of the rat glucocorticoid receptor (GR). Generally, any TF fused to the GR is retained in the cytoplasm in the absence of a synthetic glucocorticoid hormone, e.g. dexamethasone (DEX), due to physical interaction with the heat shock protein 90 (HSP90) complex. Application and binding of DEX lead to the dissociation of the HSP90 and GR, and thus “activates” the TF-GR fusion protein by allowing it to enter the nucleus, where the TF can activate or repress the expression of its target genes (Fig. 1; Craft et al. 2005; Samalova et al. 2005; Yamaguchi et al. 2015). First reports on the potential applicability of the GR system in plants date back to 1991 (Schena et al. 1991) and later GR based systems were widely used in *Arabidopsis* research. They are characterized by minimal GR-TF protein activity in the absence of DEX, rapid activation upon DEX application and the apparent neutrality of DEX itself (at concentration used by researchers) toward plant development and phenotype (Craft et al. 2005; Yamaguchi et al. 2015; Samalova and Moore 2021).

TFs are key regulators of eukaryotic gene expression and in *Arabidopsis* approximately 2000 genes encoding TFs have been recognized (https://agris-knowledgebase.org/AtTFDB). It is thus not surprising that posttranslational TF activation system, in which the TF under investigation is directly fused to the GR and expressed from a promoter of choice, has been a powerful and widely used experimental approach for deducing TFs’ mode of action (Fig. 1). As GR induction can be combined with the application of cycloheximide (a protein biosynthesis inhibitor) the immediate, direct targets of the TF can potentially be identified (Yamaguchi et al. 2015). GR-based induction is particularly valuable for TFs whose overexpression is lethal or causes very strong phenotypic changes from early stages of plant development, as DEX can be applied at the desired stage of growth, allowing the identification of signaling pathways affected by a particular TF in plants that initially appear wild-type.

GR fusions were thus used, throughout the years, to deduce the role (and direct targets) of various key developmental TFs, such as *WUSCHEL* and *WOX5* – involved in shoot and root apical meristem maintenance, respectively (Yadav et al. 2011; Pi et al. 2015; Ma et al. 2019), *VND6* and *VND7* – involved in xylem vessel formation (Yamaguchi et al. 2010), or *REV* and *KAN1* – involved in adaxial/abaxial domain specification (Reinhardt et al. 2013; Xie et al. 2015). In research, TF-GR fusion proteins have been mostly expressed from a constitutive promoter (e.g. *CaMV35S*, *UBQI10*), but tissue-specific promoters can also be employed. Interestingly, *CaMV35S* promoter was, for example, used in lately established collection of 1255 DEX inducible TF-GRs from *Arabidopsis* (ecotype Columbia-0), a valuable research resource that shows high coverage in a broad range of TF families (Shimada et al. 2022).

**Fig. 2:**
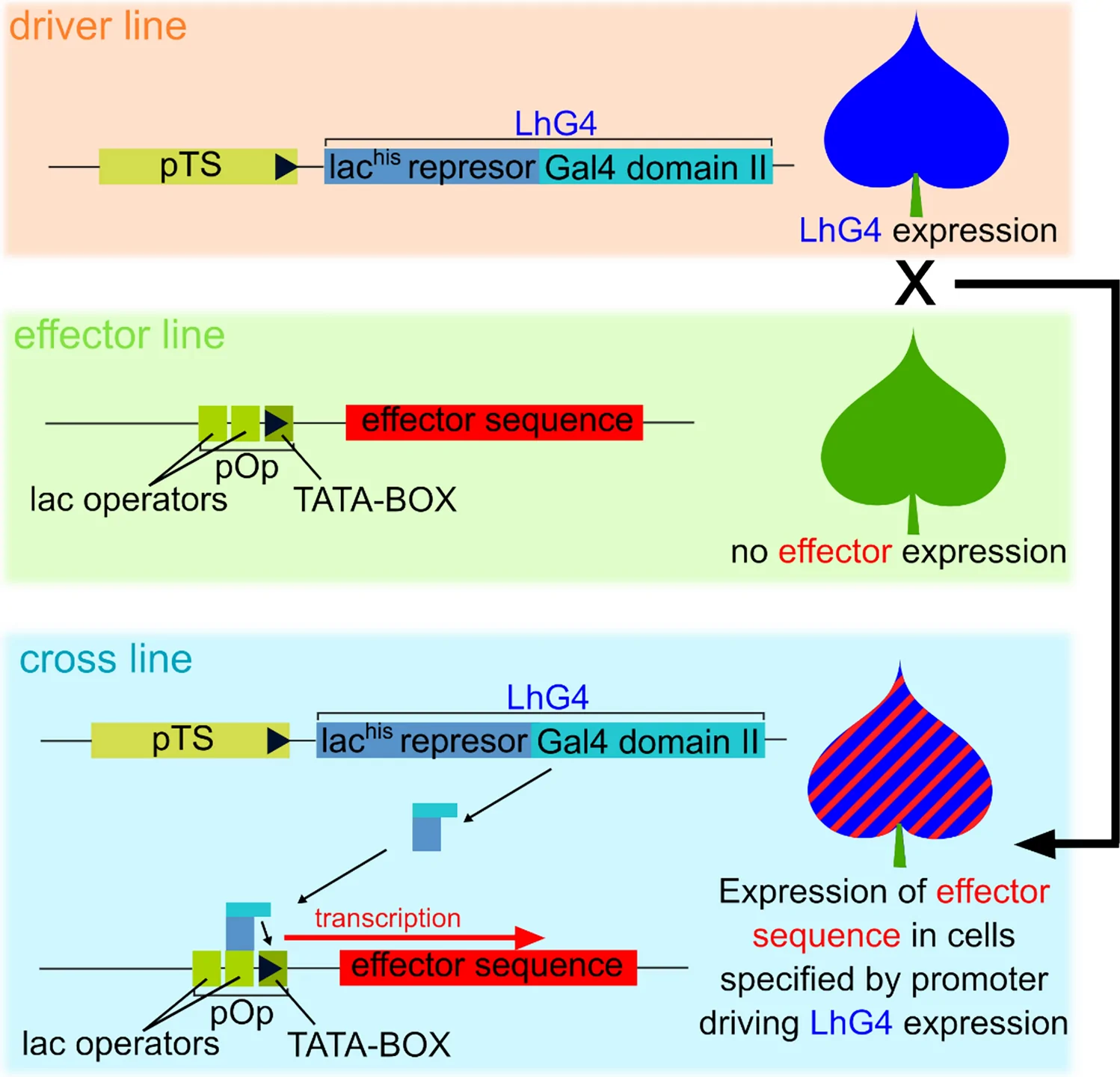
Classical pOp/LhG4 system. pOp/LhG4 requires two transgenic lines to be crossed: a driver and an effector. Driver plants contain a construct that enables expression of LhG4 from a tissue specific (pTS) promoter of choice (e.g. that would drive the expression in the whole leaf lamina, as marked by dark blue color). Effector lines contain a desired effector sequence under the control of a synthetic promoter pOp (LhG4 target promoter). After crossing both lines, transcription of the effector sequence (marked with red color) will take place only in cells in which the promoter controlling LhG4 expression is active (marked as red lines in leaf lamina). Figure prepared using Inkscape version 1.4.2

However, a direct fusion approach cannot be employed for proteins functioning in cellular compartments other than the nucleus and it may limit the possibility of adding a fluorescent tag to track the spatial correctness and strength of TF expression. Moreover, the GR tag is a large globular domain which may alter the TF structure and/or its physical properties (for example, by masking crucial functional domains, blocking protein–protein or protein-DNA interactions) and, consequently, a TF function (Picard 2000; Samalova et al. 2005). Therefore, a temporal control over transgene expression via GR was integrated with the two-component pOp/LhG4 system. The classical pOp/LhG4 system consisted of two components with the first one being the pOp promoter (two lac operators upstream of a *CaMV 35S* promoter) driving the expression of a selected effector gene. The second one was the chimeric transcription factor LhG4 (comprising a transcription-activation domain-II from Gal4 of *Saccharomyces cerevisiae* and high DNA-binding affinity mutant of the *Escherichia coli* lac repressor) expressed from a promoter of choice (Moore et al. 1998; Rutherford et al. 2005). After combining the two components in a single plant, the binding of the pOp promoter by LhG4 activates (via the Gal4 domain) transcription of the genes of interest cloned downstream of the pOp promoter sequence (Fig. 2). Their expression occurs in cells expressing LhG4, with LhG4 expression controlled by a promoter of choice (Fig. 2) (Moore et al. 1998; Rutherford et al. 2005). In this way, the pOp/LhG4 system provides spatial control over transgene expression.

The GR-LhG4/pOp system was established by providing a temporal control to the two-component pOp/LhG4 system by fusion of LhG4 with the ligand-binding domain (LBD) of GR, which proved to be functional for both *Arabidopsis* (Craft et al. 2005) and *Nicotiana tabacum* (Samalova et al. 2005). As in the TF-GR system described earlier, the GR-LhG4 fusion protein, in the absence of the ligand (inductor), is sequestered in an inactive complex due to the interaction between GR and HSP90. Only after application of the inductor (DEX), which mediates dissociation of the fusion protein from HSP90, can GR-LhG4 translocate to the nucleus, bind the pOp promoter and induce expression of the target genes (Craft et al. 2005; Samalova et al. 2005). In the first *Arabidopsis* study LhGR was expressed from the ubiquitous *CaMV35S* promoter to trigger the expression of the *GUS* reporter gene and to induce ectopic *IPT* (*ISOPENTENYL TRANSFERASE*) gene expression (Craft et al. 2005). Transgenic plants were treated with DEX concentrations ranging from 0.01 μM to 100 μM, and 10 μM and 20 μM concentrations were recommended for further use. To create an optimal LhGR construct, different GR positions relative to the *lac* repressor and the Gal4 activation domain were tested: the carboxy terminus (LhGR-C), the amino terminus (LhGR-N) and an internal position (LhGR-I) (Craft et al. 2005). All types of GR fusions were DEX-inducible, but LhGR-N was proved superior, as the LhGR-C and LhGR-I constructs were prone to leakiness, while the LhGR-I construct was additionally less active (Craft et al. 2005).

#### Experimental approaches and resources

**Fig. 3:**
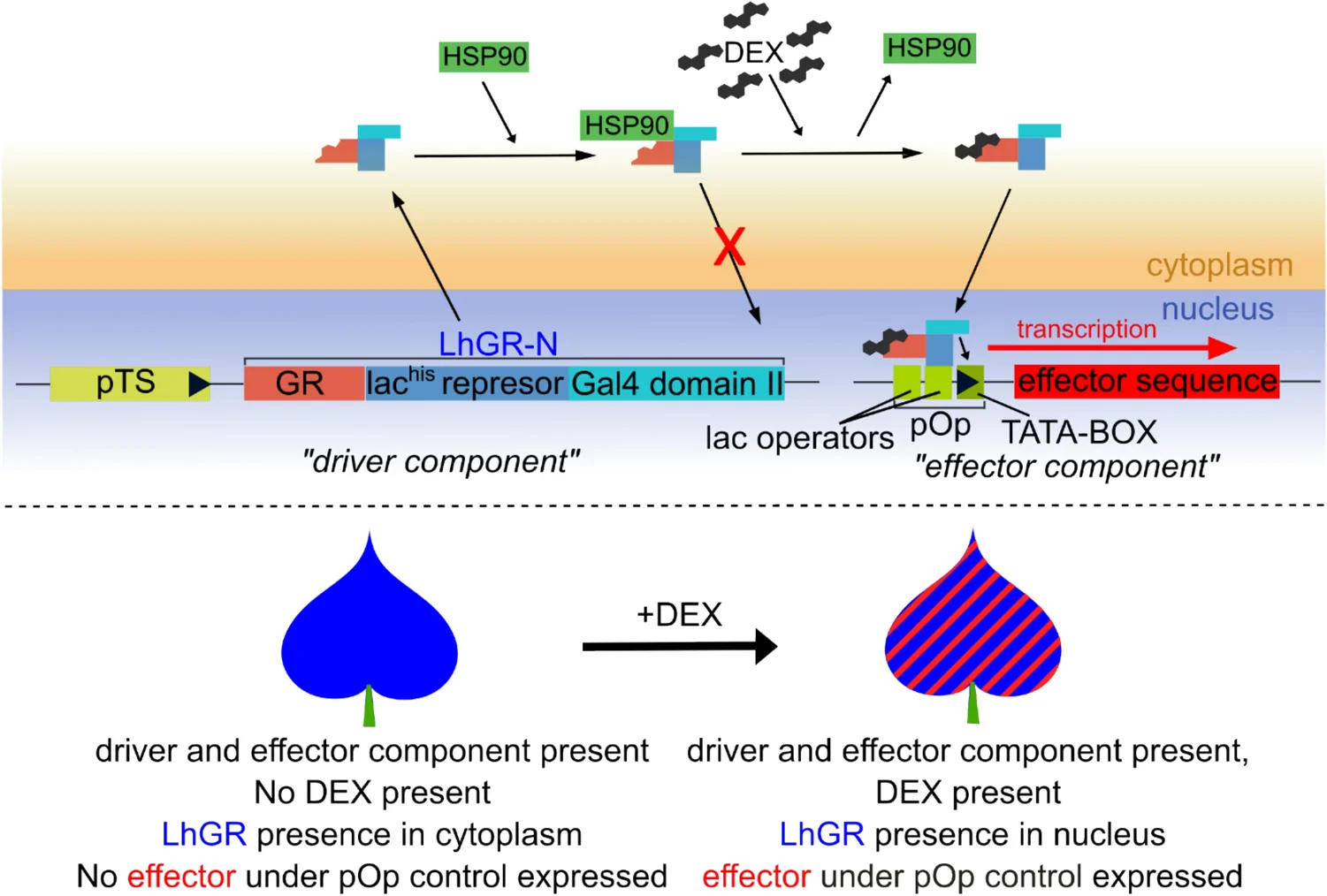
GR-LhG4/pOp mode of action. The system requires the presence of two components in a cell: a driver and an effector. The driver enables expression of LhGR (LhG4 fused to GR) from a tissue specific (pTS) promoter of choice (e.g. that would drive the expression in the whole leaf lamina, as marked by dark blue color in lower panel). The effector contains a desired effector sequence under the control of a synthetic pOp promoter. Without DEX presence, LhGR is expressed (marked in blue color in leaf lamina) but, due to binding by the HSP90 protein, cannot activate transcription of an effector sequence from its cognate pOp promoted. Upon DEX application HSP90 detaches from the GR, enabling LhGR to translocate to the nucleus, and to activate transcription of effector sequences (marked by red lines in leaf lamina in the lower panel). Expression of the effector sequence will have the same pattern as expression of LhGR. Figure prepared using Inkscape version 1.4.2

The two-component GR-LhG4/pOp systems consist of a “driver”, in which expression of GR-LhG4 (N-terminal fusion) is controlled by a tissue-specific promoter (pTS) selected by the researcher, and an “effector” in which a transgene of interest (effector) is under the control of the pOp promoter (Fig. 3). To date, various experimental designs have been implemented (for available vector backbones that can be easily utilized and/or technical notes on inductor applications see e.g. Karimi et al. 2007; Borghi 2010; Samalova et al. 2019; Zerin et al. 2023). In some cases, the vector for plant transformation contained driver (sometimes also called activator) and effector components combined (e.g. *pRPS5A:LhGR* > *pOp:EXPA1:mCherry* in Samalova et al. (2024)). In others, already generated driver lines were transformed with the effector vector (e.g. *pAtUBQ10::LhGR* driver line transformed with the *pOp:miR165a* vector in Sinha et al. (2023)), or driver and effector lines were created separately and then crossed together for the analyses performed in following generations (e.g. *pTS:LhGR pOp:mTurquoise2* driver lines crossed with the effector line *pOp:VND7-VP16* in Schürholz et al. (2018)). The first approach appears to be the fastest; however, in comparison with the latter two, it does not allow researchers to take advantage of existing resources (e.g. driver lines). In all three approaches, reporter genes are often incorporated into the system as a highly convenient method for tracing induction over time and space. *GUS* or fluorescent reporter genes can be expressed from the pOp promoter (e.g*. pOp:GUS* in Blanvillain et al. (2011) or *pOp:mTurquoise2* in Schürholz et al. (2018)), or fluorescent proteins can be fused directly to the target protein (e.g. described above *pRPS5A:LhGR* > *pOp:EXPA1:mCherry* in Samalova et al. (2024)). The presence of a reporter gene in the GR-LhG4/pOp system also has practical implications. It simplifies the process of selecting new transgenic lines (including those derived using the GR-LhG4/pOp system) after transformation. For example, it can be used to confirm the presence and specificity of transgene expression, to compare the level of transgene expression between several independent transformant lines and, in case of induction systems, additionally to test for potential baseline leakiness.

The GR-LhG4/pOp system was used to decipher the role of various TFs, such as zinc finger TFs *GIS2* (*GLABROUS INFLORESCENCE STEMS 2*) and *ZFP8 (ZINK FINGER PROTEIN 8)* in trichome development (Yasin et al. 2025), homeodomain TF *WOX5* (*WUSCHEL-RELATED HOMEOBOX 5*) in root stem cells maintenance (Pi et al. 2015) or GARP TF *GLK* (*GOLDEN2-LIKE*) in photosynthetic genes regulation (Waters et al. 2009). Furthermore, this system was also used in analyses of genes not being TFs. For example, to decipher how *EXPA1 (EXPANSIN 1)* impacts cell wall dynamics, shoot architecture and drought tolerance (Balkova et al. 2025) or how a 25-amino-acid peptide KISS OF DEATH (KOD) activates a PCD pathway (Blanvillain et al. 2011). GR-LhG4/pOp facilitated conditional silencing of selected genes, such as *AtCTF7 (CHROMOSOME TRANSMISSION FIDELITY 7)* silenced via RNAi (Singh et al. 2013), or *AtPECT1 (ETHANOLAMINE-PHOSPHATE CYTIDYLYTRANSFERASE 1)* via artificial micro RNA (Nakamura et al. 2014), or to analyze the consequences of hormone overproduction (e.g. cytokinin via *IPT* gene inducible misexpression (Craft et al. 2005; Kuderová et al. 2008; Lochmanová et al. 2008; Černý et al. 2013; Schäfer et al. 2013)). The system has proven to be a powerful tool enabling synchronization of various transgenes expression, allowing changes in a wide range of developmental processes to be monitored in precisely selected tissues and stages of plant growth, across various levels of organization (from the organismal, through the tissue and cellular to the molecular level).

In consequence of a broad use of this experimental approach up to now, many GR-LhG4/pOp transgenic *Arabidopsis* plants (driver and effector lines separately, and with driver and effector parts combined) with various promoters driving the GR-LhG4 expression have been developed and utilized (see Table 1 for a description of published *Arabidopsis* transgenic lines). Many researchers already utilize pOp/LhGR vectors (or transgenic lines) developed and extensively characterized in Craft et al. (2005) and Samalova et al. (2019). GR-LhG4/pOp has, therefore, the potential to become the prime induction system in *Arabidopsis* research, which, together with a general trend of data and resource sharing within the plant community, could further accelerate plant research.

**Table 1:** Resource table depicting available transgenic lines (and DEX application procedures) from publications that use LhGR/pOp system in *Arabidopsis* plants

Resource	DEX application procedures	Ref
- for promoters’ exact specificity and full names of genes see reference articles - for simplicity and clarity genetic constructs description was standardized and does not fully resemble original articles styles		
*-CaMV35S:LhG4 with N-, I- or N-terminal GR* *-pOp:GUS* *-pOp:ipt* *-pOp1:GUS* *-pOp2:GUS* *-pOp4:GUS* *-pOp6:GUS* *-pOp6:ipt* And combination of various driver and effector lines	Analysis of roots, whole seedlings, inflorescences, leaves: Seedlings grown for 14 or 17 days on solid MS medium with various DEX concentrations (0.1; 10; 20 µM DEX) Seedlings grown for 7 days on solid MS medium, then transferred to medium with DEX (concentration not specified) for 14 days Several-week-old soil grown plants watered with 20 µM DEX every 2–3 days for 3 weeks or for 1 week Inflorescences of plants cut and placed to 20 µM DEX for 6; 12; 24 h Rosette leaves of 5-week-old plants painted with 20 µM DEX Around 2-week-old plants grown for 48 h in liquid medium and then induced with liquid medium with 1 nM, 10 nM, 0.1µM, 1 µM, 10 µM DEX	Craft et al. 2005
*-p35S:LhGR* > *pOp:ipt (*Craft et al. 2005*)*	Analysis of whole seedlings: Seedlings grown in liquid MS media with 10 μM DEX for 2 days Analysis of leaves: Plants grown on solid MS medium for 1 week, then transferred to solid MS medium with 10 µM DEX for 2 weeks 10 day-old plants sprayed with 10 µM DEX for 2 h Plants grown for 10 days on solid MS medium with 1 µM DEX	Jasinski et al. 2005
*-p35S:LhGR* *-p35S:LhGR* > *pOp:CLV3dsRNAi* (also with markers: *pCLV3::mGFP5-ER* and* p35S:YFP29-1PMlocalised)*	Analysis of SAM: Plants grown on solid MS medium, then a drop of 10 µM DEX placed on SAM upon bolting (12 h or 24 h intervals) Seeds germinated on solid MS medium with 10 µM DEX, then seedlings transferred to soil and watered with 10 µM DEX every alternate day until bolting, for 7; 16; 18 or 30 days	Reddy and Meyerowitz 2005
*-p35S:LhGR (*Craft et al. 2005) *-pOpOff2::PDS* *-pOpOff2::PDS* *-pOpOff2(kan):LUC* *(pOpOff1 vector—pOp6 promoter drives expression of a hpRNA cassette derived from* *pHELLSGATE12 and a GUS reporter gene)*	Analysis of whole seedlings: Plants grown on solid MS medium for 12 days, transferred to medium with 10 µM DEX for 5 days, and analyzed after 6 h and then every 24 h	Wielopolska et al. 2005
*-p35S:LhGR* *-p35S:LhGR* > *pOp:WIN1-HA*	Analysis of seedlings: Seedlings grown on MS solid medium for 3 or 7 days, then immersed with 20 µM DEX for 2–5 days Analysis of leaves: Long-term induction: plants (including leaves) sprayed every 3 days from cotyledon stage with 10 µM DEX for several weeks Temporary induction: 3 week-old plants sprayed once with 10 µM DEX	Kannangara et al. 2007
*-p35S:LhGR* > *pOp:ipt* *(*Craft et al. 2005*)*	Analysis of whole seedlings: Seedlings germinated and grown on MS solid medium with 70 nM, 200 nM, 500 nM DEX or seedlings grown on MS solid medium then transferred to solid medium with 500 nM DEX Analysis of siliques: Inflorescence and siliques dipped in 1; 5 and 25 µM DEX for 5 s every 2 days until siliques reached stage 18	Souček et al. 2007
*-p35S:LhGR* > *pOp:ipt (*Craft et al. 2005*)* (also with* DR5:GUS)*	Analysis of roots: Plants either germinated on medium with 10 μM DEX for 24 h or germinated on solid MS medium and then transferred (at various age) to DEX-containing medium (10 μM) for 24 h	Kuderová et al. 2008
*-p35S:LhGR* > *pOp:ipt* (Craft et al. 2005)	Analysis of whole seedlings: Seedlings germinated and grown on MS solid medium with DEX (0.1; 0.25; 0.5; 1 µM)	Lochmanová et al. 2008
*-p35S:LhGR (*Craft et al. 2005*)* *-p35S:LhGR* > *pOp:MAX4 (with GUS)*	Analysis of whole plants: Plants grown on F2 + sand for 8 weeks, then 1 mM DEX in lanolin paste applied either encircling the hypocotyl or unilaterally; branching analysis after 4.5 weeks	Ongaro et al. 2008
-*35S:LhGR* > *pOp:FLC (with GUS)*	Analysis of embryos: Soil grown flowering plants dipped in 10 µM DEX Analysis of seedlings (mostly cotyledons): Seedlings grown on solid MS medium with 10 µM DEX	Sheldon et al. 2008
*-p35S:LhGR (*Craft et al. 2005*)* *-p35S:LhGR* > pOp:*AtSCC2-RNAi*	Analysis of generative plants (flowers, pollen, ovules, embryos): 4 week-old bolting plants subterraneously irrigated with 20 µM DEX	Sebastian et al. 2009
*-p35S:LhGR (*Craft et al. 2005*)* *-p35S:LhGR* > *pOp:GLK1-FLAG* *-p35S:LhGR* > *pOp:GLK2-FLAG* (in *glk1 glk2* double mutant background)	Analysis of whole seedlings: 10 day old plants transferred from solid MS medium to liquid MS medium, after 48 h seedlings treated with 10 µM DEX	Waters et al. 2009
*-pML1:LhGR* *-pML1:LhGR* > *pOp:TagRFP-T-MAP4*	Not specified	Heisler et al. 2010
*-p35S:LhGR* > *pOp:ipt (with GUS)*	Plants grown on sterile filter paper on MS plates for 10 days, then transferred to plates with 10 µM DEX for 2; 4; or 6 days; analysis of seedlings	Jeon et al. 2010
*-p35S:LhGR* > *pOp:KOD:GFP (with GUS)* *-p35S:LhGR* > *pOp:BAX:YFP (with GUS)*	Dissected leaves of seedlings submerged via petioles in 30 µM DEX for 3–4 days for necrosis evaluation 14 day-old plants sprayed with 20 µM DEX (just before drought stress initiation) and on the 5th and 10th day of the stress progression; analysis of leaves	Blanvillain et al. 2011
*-p35S:LhGR* > *pOp:ASH2R (point mutated version of ASH2R: s T*_*136*_* to A and K*_*187*_* to R)* *-p35S:LhGR* > *pOp:ash2r-1*	Analysis of shoot apices: Seedlings grown (method not specified) for 7 days and then treated (method not specified) once with 10 µM DEX for 72 h Seedlings grown (method not specified) for 7 days and then treated (method not specified) with 10 µM DEX twice (second one after 36 h) then shoot apices with newly emerged leaves harvested after 24; 48; 72; 96 h	Jiang et al. 2011
*-p35S:LhGR* > pOp:PHB* (dominant miR insensitive version of PHB)	Analysis of roots: Seedlings germinated and grown on MS solid medium with 50 µM DEX for 7 days Seedling germinated on MS solid medium then treated with 50 µM DEX for 4 h (application not specified)	Dello Ioio et al. 2012
*-pTobRB7:LhGR* > *pOp:hpGF (with GUS)* (with* 35S:GFP)* *(hpG F- hairpin RNA targeting and thus silencing GFP)*	Analysis of roots, hypocotyl, stem at various stages: Plants grown on solid MS medium with 10 µM DEX or transferred to solid MS medium with 10 µM DEX (at various developmental stages) Plants grown on medium with 10 µM DEX transferred to soil (and drenched from DEX)	Liang et al. 2012
*-p35S:LhGR* > *pOp:ipt (*Craft et al. 2005*)* *-p35S:LhGR* > *pOp:HvCKX2*	Analysis of whole seedlings: Seedlings grown on MS solid medium for 7 days, then transferred to solid medium with 20 µM DEX for 6; 12; 24; 48 h	Černý et al. 2013
*-p35S:LhGR* > *pOp:ARR22-HA (with GUS*) *-p35S:LhGR* > *pOp:ARR22*^*D74N*^*-HA* (*with GUS*) *(ARR22*^*D74N*^*—putative Asp residue phospho-accepting site at amino acid number 74 mutated to inert Asn residue)*	Analysis of seedlings: Plants grown on sterile filter papers for 12 days, then transferred for 6 h to MS solid medium with 10 μM DEX Plants grown on MS solid medium for 9–12 days, then incubated in MS medium with 10 μM DEX for 6 h, 48 h or 5 days Plants grown for 7 days in soil, then sprayed with 10 μM DEX once per 2 days (for total 6 days) and analyzed after 12 days or 3 weeks Plants grown for 7 days on Whatman filter placed on MS solid medium plates, then transferred to MS solid medium with 10 μM DEX for 7 days	Kang et al. 2013
*-p35S:LhGR* > *pOp:EXP17RNAi*	Analysis of roots: Plants grown on Whatman filter paper on top of agar plates, filter paper with the seedlings then transferred to a plate with 10 μM DEX for 2; 4 h	Lee and Kim 2013
*-p35S:LhGR* > *pOp:AG-amiRNA*	Analysis of inflorescence (including flowers): Plants grown on soil, then inflorescences treated (method not specified) with 10 µM DEX	Ó’Maoiléidigh et al. 2013
*-p35S:LhGR (*Craft et al. 2005*)* *-p35S:LhGR* > *pOp:AtCTF7-RNAi*	Analysis of seedlings: 7 day old seedlings transferred to solid medium with 20 μM DEX for 7 days; Analysis of siliques (including ovules) and anthers: Adult, flowering plants watered by subirrigation with 20 μM DEX and analyzed after 5 days	Singh et al. 2013
*-p35S:LhGR* > *pOp:PLAT1 (with GUS)*	Analysis of seedlings: 6 day-old seedlings transferred from solid MS medium to MS solid medium with 5 µM DEX and for 4 days	Hyun et al. 2014
-*p35S:LhGR* > *pOp:HvCKX2 (*Černý et al. 2013)	Analysis of hypocotyls: Seedlings grown on MS solid medium with 20 µM DEX for around 7 days	Černý et al. 2014
*-pFWAL:LhGR* > *pOp:NLS-mCherry-P2A-DN-ACTIN* *(DN-ACTIN—dominan negative form of ACTIN8)*	Analysis of fertilization after pollination: Mature emasculated pistils submerged in 40 µM DEX	Kawashima et al. 2014
*-p35S:LhGR* > *pOp:PECT1-amiRNA*	Analysis of SAM: Plants sprayed with DEX (concentration not specified) twice daily for 10 days	Nakamura et al. 2014
*-p35S:LhGR* > *pOp:AG-amiRNA (*Ó’Maoiléidigh et al. 2013*)* *-p35S:LhGR* > *pOp:AP3-amiRNA*	Analysis of whole seedlings: 7 day-old seedlings grown on MS solid medium then transferred to MS liquid medium with 1 µM DEX Analysis of inflorescence: 4 week-old plants treated (method not specified) with 10 µM DEX (inflorescence including flowers) daily for one week or for 24 h	Ó’Maoiléidigh et al. 2015
-p35S:LhGR > pOp:WOX5-YFP (with GUS)	Analysis of roots: 5 day-old seedlings treated (application method not specified) with 10 mM DEX	Pi et al. 2015
*-p35S:LhGR* > *pOp:RAB-A5c (with GUS)* *-AtRPS5A:LhGR* > *pOp: RAB-A5c [N125I]* *(with GUS)* *(RAB-A5c[N125I]—GTPase carrying Asn-Ile substitution in the nucleotide-binding pocket)*	Analysis of seedlings (mostly roots): Seedlings grown on MS solid medium, after 5 days transferred to MS medium with 20 µM DEX for various time points (up to 14 days) Seedlings germinated and grown on MS solid medium with 100 nM, 300 nM, 20 µM DEX for up to 7 days	Kirchhelle et al. 2016
*-pML1:LhGR* *-pML1:LhGR* > *pOp:VENUS-HAT3* *-pML1:LhGR* > *pOp:REVr-2xVENUS* *-pML1:LhGR* > *pOp:REVr-2xVENUS* (combined with *pMIR165a:mTagBFP-ER* or *pMIR165a::BFP-ER* and* pMIR165a(-cis):mTagBFP-ER) (in hat3 athb4 mutant background)* *-pUBQ10:LhGR* > *pOp:MIR165a* (combined with *pMIR166a:GFP-ER* and *pHAT3:VENUS-HAT3* or* pATHB4:VENUS-ATHB4)*	Analysis of seedlings: Seedlings grown on solid GM medium with 10 μM DEX for 3; 4 or 5 days Analysis of shoot apices (sometimes also young leaves): 7 day-old seedlings grown on solid GM medium transferred to 10 µM DEX for 3 days 10 day-old seedlings immersed in 20 µM DEX for 30 min, then to 20 µM CHX solution supplied with 10 µM DEX for 3 h 10 day-old seedlings immersed in 10 µM DEX for 3 h	Merelo et al. 2016
*p35S:LhGR* > *pOp:ipt (*Craft et al. 2005*)*	Analyses of roots and leaves, also in relation to temperature shifts: Seedlings cultivated hydroponically, then 20 µM DEX was applied to the hydropony medium	Skalák et al. 2016
*-p35S:LhGR* > *pOp:*GFP-DIG1 *-p35S:LhGR* > *pOp:*GFP-DIG2 *-p35S:LhGR* > *pOp:*GFP	Analysis of seedlings: Hydroponic-grown seedlings treated with 500 nM DEX for 10 days Seedlings grown on LS solid medium with 200 nM DEX for 8–9 days	Song et al. 2016
*-pUBQ10:LhGR* > *pOp:STM MIR165/166* *-CLV3:LhGR* > *pOp:KAN-2xGFP* *-ATML1:LhGR* > *pOp:KAN-GFP* *-ATML1:LhGR* > *pOp:REVr-2 VENUS -(MIR165/166-resistant REV2)* *-ATML1:LhGR* > *pOp:PHVr (MIR165/166-resistant PHAVOLUTA)*	Analysis of SAM: Plants germinated and grown on GM medium with 10 µM DEX for 4 days 10 µM DEX applied (method not specified) to inflorescence apices every second day for three times	Caggiano et al. 2017
*-*p35S:LhGR > *pOp:LEC1*	Analysis of aerial parts of seedlings: Seedlings grown on solid MS medium for 7 days, then treated (method not specified) with 10 μM DEX twice (second application after 36 h) Analysis of germination: Seeds soaked in 10 μM DEX for 1 h, then put on solid MS medium for 2 or 3 days	Tao et al. 2017
*-INDp:LhGR* > *pOp:IND (with GUS)* (in wildtype and *ind-2* mutant plants)	Analysis of siliques: Inflorescence and siliques dipped in 10 µM DEX for 5 s every 2 days for several days	Li et al. 2018
*-p35S:LhGR* > *pOp:HvCKX2 (*Cerný et al. 2013*)* *-p35S:LhGR* > *pOp:ipt (*Craft et al. 2005*)*	Analysis of leaves: 14 day-old plants (including leaves) sprayed with 20 µM DEX (just before drought stress initiation) and on the 5th and 10th day of the stress progression	Prerostova et al. 2018
- *pTS:LhGR* > *pOp:SP-mTurquoise2-HDEL* (pTS – tissue specific promoters: *pATHB8; pXPP, pAHP6; pPXY, pTMO5; pSMXL5; pSCR, pCASP1; pVND7; pAPL, pWOX4; pNST3; pLTP1; pAT2G3830; pML1; pCLV3; pREV, pUFO, pCUC*) *-pSCR:LhGR* > *pOp:SP-mVenus- HDEL* -*pCLV3:LhGR* > *pOp:3xGFP-NLS* *-pSCR:LhGR* > *pOp:ND7-VP16*	Analysis of whole seedlings or roots: Seedlings grown on solid medium with 0.1; 1; 10; 100 µM DEX for 5 days after germination Analysis of shoot apices: Inflorescence shoots dipped in 10 µM DEX and analyzed after 24 h Analysis of inflorescence: Inflorescence stems sprayed with 10 µM DEX and analyzed after 48 h	Schürholz et al. 2018
*-pIND:LhGR* > *pOp:Barnase-Barstar* (also with* WIND1::GUS)*	Analysis of siliques: Inflorescence and siliques dipped in 10 µM DEX for 5 s every 2 days for several days	Li et al. 2019
*-pPXY:LhGR* > *pOp:pro:Cre* *-pSMXL5:LhGR* > *pOp:pro:Cre* *-pWOX4:LhGR* > *pOp:pro:Cre* *-pPXY:LhGR* > *pOp:pro:Cre/35S:loxP:loxP:er-YFP* *-pSMXL5:LhGR* > *pOp::Cre/35S:loxP:loxP:er-YFP* -pWOX4:*LhGR* > *pOp::Cre/35S:loxP:loxP:er-YFP* *-pNST3:LhGR* > *pOp::Cre/35S:loxP:loxP:er-YFP* *-pAPL:LhGR* > *pOp::Cre/l35S:loxPt:loxP:er-YFP*	Analysis of seedlings (including vasculature): Seedlings germinated on solid medium, then transferred to MS medium with 10 µM DEX for 7 days Analysis of hypocotyls at different times after induction: Seedlings germinated on medium, then after 22 days tissue was tied around a hypocotyl, and 500 nM/2.5 μM DEX was applied to the paper knot Analysis of stem (including vasculature): Inflorescence (including shoot apices) of soil grown plants treated five times at 2 day intervals at various developmental stages with 10 µM DEX	Shi et al. 2019
*-pUBQ10:LhGR* > *pOp:AtSar1B1H74L-CFP* (with* VHA-a1-GFP)*	Analysis of roots: Seedlings grown on solid MS medium, then incubated in liquid MS medium with 60 µM DEX	Lupanga et al. 2020
*-p35S:LhGR* > *pOp:AP2m3 (with GUS)*	Analysis of shoot apices: Plants grown in soil until 2 weeks after bolting, then shoot apices dipped in 5 µM DEX and dissected after 6 and 8 h A drop of DEX (concentration not specified) applied to the center of the SAM of soil grown plants (age not specified)	Martínez-Fernández et al. 2020
*-pML1:LhGR* > *pOp*:*REVr-2VENUS* *(MIR165/166-resistant REV)* *-pML1:LhGR* > *pOp:KAN1-2GFP* *-pUBQ10:LhGR* > *pOp:miRNA165a* (in *ap1 cal* mutant; some with* pAtML1:mTag-BFP-ER)*	Analysis of shoot apices: 10 μM DEX applied once to the inflorescence meristem, which was collected after 6 or 16 h 10 μM DEX applied to the inflorescence every second day for total 7 days	Ram et al. 2020
*-p35S:LhGR* *-p35S:LhGR* > *pOp:TT1* *-p35S:LhGR* > *pOp:TT1-GFP* *-p35S:LhGR* > *pOp:NTT* *-p35S:LhGR* > *pOp:NTT1-GFP*	Analysis of whole seedling: 7 day-old seedlings transferred to MS liquid medium supplied with 1 µM DEX Analysis of whole plants: 5 week-old plants (including flowers, leaves, inflorescence stem) sprayed with different concentrations of DEX (0.001; 0.05; 0.1; and 1 µM) and analyzed after 4 days Analysis of leaf discs: Leaves disks collected and sprayed with 1 µM of DEX for variable time	Roldan et al. 2020
*-pDRNL:LhGR* > *pOp:CRE* *-pOp:loxP-GFP-loxP-GUS* *(and the combination with both components)* *-pOp:DT-A* *-pDRNL:LhGR* > *pOp:DT-A (diphtheria toxin A chain coding sequence)*	Analysis of cotyledons, leaves: Seedlings germinated on solid MS medium with 5 µM DEX Analysis of inflorescence: Plants grown in soil and sprayed with DEX at fully open cotyledon stage or prior to bolting at 6–7 leaf stages Analysis of embryos: Primary inflorescences sprayed with 5 µM DEX at the time of first flower opening, then seeds harvested from first 5–6 siliques for growth defects analysis	Glowa et al. 2021
*-pRPS5A:LhGR* *-pRPS5A:LhGR* > *pOp:PLL12*	Analysis of whole seedling (including vasculature): Seedlings germinated on solid MS medium, then transferred to soil and treated (method not specified) at appropriate developmental stages with 10 µM DEX Seedlings germinated on solid MS medium, then transferred to MS medium with 10 µM DEX for 7 days	Bush et al. 2022
-*pRPS5a:LhGR* > *pOp:nls3xVenus* *-pRPS5a:LhGR* > *pOp: H1.1-RFP* -*pRPS5a:LhGR* > *pOp:∆KinesinYFP* *-pRPS5a:LhGR* > *pOp:MET1-amiRNA (with GUS)* *-pRPS5a:LhGR* > *pOp:FIE-amiRNA (with GUS)*	Analysis of ovules and anthers: Singular or dual application (the additional one after around half a day) to flower buds with soft-hair paintbrush; 10 µM DEX	Schubert et al. 2022
*-pAHP6:LhGR* > *pOp:mTutquoise2 (4–1#668)* *-pXPP:LhGR* > *pOp:mTutquoise2 (D10-2001)* *-pSCR:LhGR* > *pOp:mTutquoise2 (17–0215#680)* *(*Schurholz et al. 2018*)* *-pUbi:LhGR-tPea3A* *-pOp6:SaCas9-ITS2* *-pOp6:SaCas9-ADH1* *- combination of abovementioned driver and effector lines*	Analyses of roots and whole seedlings: Seedlings germinated on DEX containing MS solid media or transferred to MS medium with DEX after 5 days; 50 and 100 μM DEX Analysis of whole plants: Plants grown on herbicide-containing medium for 2 weeks, after another 12 days plants transferred to cultivation boxes with 100 μM DEX for 21 days Analysis of shoot apices: Inflorescence apices of 15 cm-tall plants treated with 10 μM DEX (application not specified)	Gehrke et al. 2023
-*pUBQ10:LhGR* -*pUBQ10:LhGR* > *pOp:miR165a* (both in wildtype and *ap1cal* mutants)	Analysis of shoots: Singular application of 10 μM DEX on seedlings shoots or application every second day for a total of 7 days (unspecified method)	Sinha et al. 2023
*-p35S:LhGR* > *pOp:PRPS1* *-p35S:LhGR* > *pOp:PRPL4*	Analysis of leaves: Plants grown for 16 days on solid MS with 2 μM DEX Plants grown for 16 days on MS medium with 2 μM DEX Leaf discs infiltrated with 2 μM DEX for 3; 6; 24 h	Tadini et al. 2023
*-AtRPS5a:LhGR* > *pOp:secRFP-RLP4sΔECD (secRFP-RLP4sΔECD—RLP4s variant lacking ECD and fused to secreted version of RFP targeting them to the secretory pathway)* *-AtRPS5a:LhGR* > *pOp:RAB-A5cN*^*125I*^ (Kirchhelle et al. 2016) (RAB-A5cN^125I^—GTPase carrying Asn-Ile substitution in the nucleotide-binding pocket)	Analysis of roots: Seedlings germinated and grown on 250 nM oryzalin and/or 1 µM DEX Seedlings germinated and grown on 10 µM DEX, after 3 days transferred to 500 nM DEX or 1 µM DEX or 500 nM DEX with 250 nM Ory or 1 µM DEX with 250 nM Ory	Elliott et al. 2024
-*pRPS5A:LhGR* > *pOp:EXPA1* *-pRPS5A* > *LhGR* > *pOp:EXPA1-mCherry*	Analysis of roots: Seedlings germinated and grown on MS solid medium, then 20 µM DEX added for 1–6 h or 24 h Seedlings germinated and grown on MS solid medium and transferred to liquid MS medium with 20 µM DEX	Samalova et al. 2024
*-p35S:LhGR* > *pOp:HB21*	Analysis of whole plants (including shoot apices): Plants grown on soil, then drop of 10 µM DEX applied to shoot apex two weeks after bolting; whole plant analysis after 5 days, then apices harvested for further analysis	Sánchez-Gerschon et al. 2024
*-p35S:LhGR* > *pOp:WOX5-3xFlag* (with *Q1630::H2B-tdTomato*)	5 day-old seedlings grown in solid MS medium treated with liquid MS medium containing 10 µM DEX for 15 min by flooding; analysis of roots	Sharma et al. 2024
*-p35S:LhGR:pOp:NST1 (with GUS)* *-p35S:LhGR:pOp:NST3 (with GUS)*	Analysis of seedlings: Seedlings grown on MS solid medium for 3 or 7 days, then immersed with 20 µM DEX for 2–5 days Analysis of inflorescence: 4 week-old soil grown plants watered with 50 µM DEX every 3 days until plants had 8 weeks	Tamadaddi et al. 2024
*-pEPFL2:LhGR* > *pOp:gEPFL* *(with 35S:H2B-GFP; in wild-type, epfl1,2 double mutant and epfl1/* + *,2,4,6 mutant background)*	Analysis of whole seedlings: 5 day-old seedlings transferred from MS medium to 2 ml of liquid MS medium containing 10 µM DEX for 7 h	Uzair et al. 2024
*-pRPS5A:LhGR* > *pOp:EXPA1 (5–4; 8–4; *Samalova et al. 2024*)*	Analysis of seedling shoots: Seedlings germinated and grown on solid medium with 20 µM DEX for 7 days or induced with DEX for 3 h Analysis of shoots and leaves: Soil grown plants watered up to the maximum soil–water holding capacity, or manually every 2–3 days with 20 µM DEX	Balkova et al. 2025
*pTS:LhGR* > *pOp:SP-mTurquoise2* *(pTS – tissue specific promoters: pML1; pREV,* *pUFO, pLTP1; pAPL, pSCR, pTMO5; pPXY, pCASP1; pSMXL5; pATHB-8)* *(*Schürholz et al. 2018*)*	Analysis of various cell types sorted from roots (including RAM), SAM and epidermis: Plants grown on MS solid medium with 30 µM DEX and analyzed after 14 or 21 days	Barff et al. 2025
*-pML1:LhGR* > *pOp:DAO1* *-pML1:LhGR* > *pOp:TAA1*	Analysis of SAM: Treatment of apices every day from 2 or 4 weeks after bolting for 1 day or 1/2 weeks with 10 µM DEX	González-Cuadra et al. 2025
*-pUBQ10:LhGR* > *pOp::AP1*^*WT*^* (with GFP)* *-pUBQ10:LhGR* > *pOp::AP1*^*tet*^* (delete exon 5,6) (with GFP)* *(in ap1-11 mutant background)*	Soil grown 10 day-old seedlings sprayed with 10 µM DEX once a day for 10 days for analysis of leaves or once for analysis of seedlings after 2; 8 and 24 h	Xu et al. 2025
*-p35S:LhGR (4c-S5; *Craft et al. 2005*)* *-p35S:LhGR > pOp:GIS2* *-p35S:LhGR > pOp:ZFP8* *-pOp:GIS2* *-pOp:ZFP8*	Analysis of inflorescence stem: Plants with 3–5 cm high primary stems sprayed with 10 µM DEX AND ANALYZED after 2 or 4 h	Yasin et al. 2025
*-pRPS5a:LhGR* > *pOp:Cul4-amiRNA (with GUS)* *-pRPS5a:LhGR* > *pOp:AIH-amiRNA (with GUS)* (both with *pH1.1::H1.1-GFP* and *pH1.2::H1.2-ECFP; 3h1 Arabidopsis* lines*)*	Analysis of siliques (including ovules): Flower buds opened with a needle for a good exposure of the carpel, then brushed with 10 µM DEX and analyzed several days post-induction	Li et al. 2026
*-p35S:LhGR* > *pOp:JUL (in jul1 mutant background) (with GUS-GFP or 3xGFP)* *-p35S:LhGR* > *pOp:JUL-amiRNA (with GUS-GFP or 3xGFP)*	Analysis of leaves: Plants grown on Gamborg B5 medium. DEX treatment (concentration and method) not specified	Park et al. 2026

Presents the publications identified by the authors. The intention was to include all relevant works; however, it is possible that some publications are not covered

^*^(with GUS) – abbreviation applied for simplicity to mark where also GUS reporter gene is transcribed in response to DEX presence

A particularly interesting resource is a set of 19 well-characterized and stable GR-LhG4 driver lines created by Schürholz et al. (2018). In these lines, tissue/cell type specific promoters drive expression of GR-LhG4 in a multitude of locations, such as endodermis, epidermis, (pro)cambium, xylem, phloem, fibers, stem cells of the SAM and RAM and cells of the peripheral SAM zones (Schürholz et al. 2018; Table 1). The system presented in Schürholz et al. (2018) is easy to use and re-adjustable, as constructs were cloned with the Golden Gate-type GreenGate cloning system which enables fast and modular creation of large constructs (Lampropoulos et al. 2013). All vectors can be obtained from Addgene Vector Database, and upon request. Driver lines are available in NASC/ABRC seed stock centers. All driver lines contain the *mTurquoise2* reporter gene for tracking the efficiency and spatial correctness of the induction (Schürholz et al. 2018). Availability of this set of driver lines offers an opportunity to speed up research, as time- and labor-consuming processes of own driver line derivation can be omitted. These established driver lines can be transformed with personally designed “effector” constructs or crossed with new effector lines (for a comprehensive protocol for self-implementation of the available components of the system and methods see e.g. Zerin et al. (2023)). This system has potential to be the basis for the development of novel tools, such as the recently published approach for targeted ablation of specific cell types without destroying other cells/tissues named as CRISPR-Kill system (Gehrke et al. 2023). This system is a promising new framework based on modified CRISPR/Cas technique, typically used for gene editing. Generally, in CRISPR/Cas-based technique, a single-guide RNA (sgRNA) directs the Cas9 endonuclease to a specific (complementary) target DNA locus, which is subsequently cleaved. The generated double-strand breaks (DSB) at the target DNA sequence are then repaired by homology-directed repair (HDR) or non-homologous end joining (NHEJ) (Bortesi and Fischer 2015). In the CRISPR-Kill system, multiple double-strand breaks are induced in conserved, repetitive genomic regions, causing death of the targeted cells (Gehrke et al. 2023). This system was created by combining driver lines developed by Schürholz et al. (2018) and newly established effector lines composed of two elements: SaCas9 nuclease (derived from *Staphylococcus aureus*) downstream of the *pOp6* promoter and the respective guide RNA (gRNA) downstream of a constitutive *U6-26* promoter (Gehrke et al. 2023). DEX application simultaneously activates the expression of the mTurquoise2 reporter, marking the target tissue, and triggers DNA breaks resulting in the target cell death.

#### Dexamethasone application

DEX is a highly potent and versatile inductor that can easily reach any target cell type within the *Arabidopsis* body, including those that are seemingly less straightforward to work with (see Table 1 for DEX application procedures and analyzed plant organs). Ovules and embryos serve as examples of structures that are generally difficult to study due to their inaccessibility. Moreover, conventional approaches using gain or loss of function mutant lines may be strongly impeded by pleiotropic effects accumulating over time or even possible detrimental effects on viable seeds production. The GR-LhG4/pOp system offers a good solution, as transgenic lines without DEX application morphologically and functionally fully resemble WT plants and produce viable seeds and can thus be successfully derived and subsequently analyzed at late stages of *Arabidopsis* development. DEX seems to be a very convenient inductor that has been delivered to the reproductive organs in various ways. In work carried out by Singh et al. (2013), DEX was applied to flowering plants via soil irrigation to decipher the role of *AtCTF7* in sister chromatid cohesion during meiosis. To study the *FLC* gene during embryo development (Sheldon et al. 2008) and processes relevant to fruit opening and dehiscence (Li et al. 2018, 2019), inflorescences and/or siliques were dipped into the DEX solution. However, to limit nonspecific effects, a more specific and less invasive procedure has recently been developed. DEX was directly applied to *Arabidopsis* carpels via a paintbrush, which allowed the researchers to activate various transgenes (e.g. *pRPS5a:LhGR* > *pOp:∆KinesinYFP* and *pRPS5a:LhGR* > *pOp:amiFIE*) only in ovules (Schubert et al. 2022). This procedure had no negative impact on the development of ovule primordia or spore mother cells (SMCs), proving that GR-LhG4/pOp system can be finetuned for small plant structures and that DEX is indeed a versatile inductor that can be applied very locally (Schubert et al. 2022).

The cambium and secondary xylem are other examples worth mentioning, in which GR-LhG4/pOp was used to successfully induce transgenes in deeply located and otherwise difficult-to-access tissues. In Bush et al. (2022), GR-LhG4/pOp-based overexpression triggered by DEX application via inflorescence stems indicated that the *PLL12* gene (*PECTIN LYASE-LIKE 12*) plays a role in cambium and xylem development and suggested the involvement of a cell wall-derived signal in the regulation of vascular differentiation (Bush et al. 2022). Tamadaddi et al. (2024), on the other hand, analyzed the role of *AtNST (NAC SECONDARY WALL THICKENING PROMOTING FACTOR)* genes in the formation of secondary cell wall (SCW) patterning in diverse cell types and developmental stages in the hypocotyl and stem; and here DEX was applied via soil irrigation (Tamadaddi et al. 2024). In Shi et al. (2019) cell lineage tracking system combining LhGR/pOp and Cre/Lox-P was developed and used to trace the fate of cells located in various distal cambium domains. In this approach DEX was applied to hypocotyls very locally via paper knots (Shi et al. 2019).

Depending on the study design, the organ analyzed, and/or the stage of growth, the inductor DEX can thus be applied to the plant in various ways in the two-component GR-LhG4/pOp system (see Table 1 for an overview and Samalova et al. (2019) and Zerin et al. (2023) for detailed dexamethasone application protocols and practical considerations). The most frequently used DEX concentrations have been 10 µM and 20 µM, although lower or higher concentrations have also been applied. Medium supplementation approaches are particularly suitable for analyzing roots and young seedlings and are straightforward to perform. To induce the system in older plants, methods for DEX administration included dipping whole shoots and spraying or watering plants with a DEX solution. Direct and specific application of DEX to target organs or tissues (e.g. with a paintbrush or a paper knot) has been less commonly used but has been shown to be effective for local induction (see e.g. Shi et al. 2019; Schubert et al. 2022; Li et al. 2026). The duration of DEX incubation ranged from hours to days to weeks, and for longer incubation periods DEX was often applied multiple times at intervals of varying duration. DEX can thus easily reach any target cell type within the *Arabidopsis* body. However, such variability in induction methodology can also cause problems of data comparison from published results.

Notably, even though spatial control is mostly achieved by the promoter used to drive the GR-LhG4 expression, to some extent it can be also achieved through the localized application of the inducer. However, DEX can also migrate to distant organs (for example, root uptake after DEX supplementation to the soil causes its acropetal transport to the stem), but, to our knowledge, its mobility within the plant body has not been thoroughly studied so far.

### GR-LhG4/pOp – systems applicability to other than *Arabidopsis* plant species

The pOp/LhGR system has been shown to be functional also in other than *Arabidopsis* plant species (see Table 2 for an overview). In *Nicotiana tabacum*, the system was implemented in the same year as in *Arabidopsis* (Craft et al. 2005; Samalova et al. 2005) and was used to express *GUS*, *LUCIFERASE* or *IPT* genes (Samalova et al. 2005). Later, GR-LhG4/pOp was used in tobacco to show that overaccumulation of cytokinin simulates stress response (Novák et al. 2013) and to study protein dynamics after blocking cellular vesicular transport from the endoplasmic reticulum to Golgi via induced SAR1-GTP overexpression (Osterrieder et al. 2010). The inducible GR-LhG4/pOp system was especially essential for the latter use, as prolonged expression of SAR1-GTP causes lethal effects on cells, which would otherwise impede the research. Tobacco plants were also used to prove that the GR-LhG4/pOp system is functional in field conditions. By adapting the protocol of DEX administration in lanolin, it was shown that *Nicotiana attenuata* transgenic plants in the field were able to effectively overexpress the *IPT* gene and to block (via RNAi approach) expression of *PDS* (*PHYTOENE DESATURASE*) (Schäfer et al. 2013).

**Table 2:** Resource table depicting available transgenic lines (and DEX application procedures) from publications that use LhGR/pOp system in non - *Arabidopsis* plants

Resource	Plant species	DEX application procedures	Ref
- for promoters’ exact specificity and full names of genes see reference articles - for simplicity and clarity genetic constructs description was standardized and does not fully resemble original articles styles			
Dicotyledenous plants			
Driver lines: * 35S-LhG4* * 35S-LhGR-N* * 35S-LhGR-I* * 35S-LhGR-C* Effector lines: *pOp:ipt-S* *pOp:ipt-660* *pOp6:GUS* *pOp6:ipt* *GUS-TMVΩ-pOp6-TMVΩ-Ipt* *GUS-TMVΩ-pOp6-TMVΩ-luc* Effector/Driver lines: * pOp-ipt-S/35S-LhG4* * pOp-ipt-S/35S-LhGR-N/I/C* * pOp6-ipt/35S-LhGR-N* * GUS-TMVΩ-pOp6-TMVΩ-Ipt/35S-LhGR-N* * GUS-TMVΩ-pOp6-TMVΩ-luc/35S-LhGR-N* * pOp6-GUS/35S-LhGR-N*	*Nicotiana tabaccum*	Analysis of seedlings: Seedlings germinated and grown on solid medium with 0.625; 1.25; 2.5; 5; 10; 20; 30 µM DEX for 4 weeks Seedling germinated and grown on solid medium with 20 µM DEX for 3; 4; 6; 12 weeks 6 week old plants’ cuttings placed in solid medium with 20 µM DEX and analyzed after 12 days Abaxial side of young/growing/fully expanded leaves of soil grown plants (age not specified) painted using paintbrush with 20 µM DEX solution and analyzed after 1; 3 and 7 days Analysis of whole plants: Auxiliary buds of soil grown plants (age not specified) painted using paintbrush with 20 µM DEX solution 3 times per week for 4 weeks 3 week-old seedling incubated in liquid medium with 20 µM DEX and analyzed after 1 h, 2 h, 4 h, 8 h, 12 h, 24 h, 36 h Plants transferred to soil and then watered with 5; 10; 20; 30; 60 µM DEX 3 times a week for 4 weeks Soil grown plants (age not specified) watered with 20 µM DEX solution 3 times per week for 4 weeks Analysis of leaves: 8 week-old soil grown plants watered with 20 µM DEX solution for 2 consecutive days or over 8-week period (not specified exactly)	Samalova et al. 2005
* -p35S:LhGR* > *pOp:Sar1-GTP; pOp-GUS* * -p35S:LhGR* > *pOp:Sar1-GTP-YFP; pOp-GUS*	*Nicotiana tabacum*	Abaxial side of leaves of soil grown plants (age not specified) painted using paintbrush with 20 µM DEX and analyzed for up to 18 h Analysis of roots: 20 µM DEX poured to form a layer on the solid medium with 10-day old seedlings, and analyzed for up to 18 h	Osterrieder et al. 2010
* -p35S:LhGR* > *pOp6:ipt* (Samalova et al. 2005)	*Nicotiana tabacum*	Analysis of whole plants: 5 week-old soil grown plants watered with 0.1; 0.25; 0.5; 1; 2; 5; 10; 20 µM DEX, and analyzed for up to 24 days	Novák et al. 2013
* -p35S:LhGR* > *pOp6:ipt* *(IPT* overexpression) * -p35S:LhGR* > *pOp6:irpds* *(PDS* silencing via RNAi)	*Nicotiana attenuata*	Analysis of whole seedlings: Seedlings germinated and grown on solid medium with 20 µM DEX (duration not specified) Analysis of whole plants: Seedlings transferred to hydroponic medium pots to which 1 µM DEX was added after 1 week and then analyzed (time not specified) Lanolin paste with 1; 5; 20; 100 µM DEX applied on the underside of leaf petiole every 3 days the earliest 3 weeks after plants germination (variable stage of growth)	Schäfer et al. 2013
* -AtRPS5a:LhGR* > *pOp6: RCO-VENUS; pOp6-GUS* *(*in *rco* mutant background)	*Cardamine hirsuta*	Analysis of whole seedlings: 14 day-old seedlings grown on solid medium, incubated in liquid medium with 10 µM DEX and analyzed after 2 h, 4 h, 6 h, 8 h, 10 h	Hajheidari et al. 2019
* -pUBQ1:LhGR* > *pOp:PHS1ΔP -mCherry* *(in p35S::GFP:TUA background)*	*Cardamine hirsuta*	Analysis of fruits: 7 mm big fruits (stage 5) with a petiole and a stem portion transferred to solid MS medium with 1 mM DEX and incubated for up to 11 days Fruits attached to stems dipped in 2 mM DEX daily for up to 10 days	Eng et al. 2026
* -p35S:LhGR* > *pOp6:hpYFP* * -p35S:LhGR* > *pOp6:hpACC* *(YFP* or* ACCase* silencing via RNAi)	*Medicago truncatula*	Analysis of roots: 8 week-old plants grown in solid medium transferred to solid media with 1; 3; 40; 50; 100 µM DEX for 8 h, 20 h, 24 h, 42 h, 4 days, and 6 days	Liu and Yoder 2016
-p*35S:LhGR* > *pOp6:RNAiACO1,pOp6:GUS* * -p35S:LhGR* > *pOp6:RNAiACO2,pOp6:GUS* * -p35S:LhGR* > *pOp6:RNAiACO,pOp6:GUS (*RNAi (construct to silence *ACO1* and *ACO2* simultaneously*)*	*Carica papaya*	Analysis of leaves: Plants (age not specified) watered 3 times during 1 day with 2 µM DEX, and analyzed after 1; 5; 9; 15; 21 days Analysis of fruits: Picked stage 2 fruits sprayed with 2 µM DEX and analyzed for up to 20 days	Sekeli et al. 2014
* -p35S:LhGR-N* > *pOp6:GUS*	*Citrange carrizo (Citrus sinensis* × *C. trifoliata)*	Analysis of whole plants: Soil grown 8 month-old plants (8 months after grafting to 4 week-old rootstock) watered twice a week with 20 µM DEX for 50; 20 or 14 days (14; 6 or 4 applications, respectively) Soil grown 3 month-old plants (3 months after grafting to 4 week-old rootstock) watered twice a week with 20 µM DEX for 14 days (4 applications)	Rossignol et al. 2014
* -p35S:LhGR* > *pOp6:AtSGR1; pOp6-GUS*	*Populus alba* × *Populus tremula*	Analysis of leaves: Around 2 month-old (30 cm high) soil grown plants sprayed with 10 µM DEX, and analyzed for up to 8 days	Ito et al. 2022
* -p35S:LhGR* > *pOp:amiR-CrSTM* * -p35S:LhGR* > *pOp:CrSTM:3* × *FLAG*	*Capsella rubella*	Analysis of seedlings: 10 day old seedlings incubated in liquid medium with 10 µM DEX and analyzed after 4 h, 8 h, 12 h Analysis of fruits: Inflorescence stems, with flowers before stage 12; dipped in 20 µM DEX and allowed to grow. The analysis of formed fruits performed at stage 13/14	Hu et al. 2025
Monocotyledonous plants			
* -pZmUbi:rcoLhGR* > *pOp6:GUS* *(rice codon optimized LhGR)*	*Oryza sativa*	Analysis of whole seedlings: Seedlings germinated and grown on solid medium with 0.01; 0.1; 1; 5; 10; 30 µM and analyzed after 12 days 3 day old seedlings grown on solid medium with 0.01; 0.1; 1; 5; 10; 30 µM DEX and analyzed after 5 days Whole 7 day old seedlings submerged in 10 µM DEX for 19 h and analyzed for up to 5 days Callus grown for 3 or 13 days on solid medium with 10 µM DEX	Vlad et al. 2019
* -pZmUbi:LhGR2* > *pOp6:YFP, pOp6:GUS* (non codon optimized for rice)	*Oryza sativa*	Various parts of plants analyzed: Seedlings are either germinated and grown on solid medium with DEX for 7–10 days, transferred to plates with DEX after 6 days of germination or submerged in liquid medium with DEX for 12–72 h. Various DEX concentrations Shoots and leaves cuttings submerged in 10–30 µM DEX for 24 h and analyzed Analysis of leaves: New leaves (both sides) of around 2 week-old seedlings painted using paintbrush with 0.01; 0.1; 1; 10; 30 μM DEX every day for two days or for 1–2 weeks every second day 4 week-old soil grown plants watered with 30 µM DEX, every day for 2 days or 1–2 weeks every second day	Samalova and Moore 2021
* -pZmUbi1:LHGR* > *pOp6:SvR1-2A-SvC1-2A- ELUC* * -pZmEf1a:LHGR* > *pOp6:SvR1 −2A- SvC1-2A- ELUC* *(rice codon optimized LhGR)*	*Setaria viridis*	Protoplasts put to medium with 10 µM or 1 µM DEX and analyzed for up to 50 h Dissected leaf discs put in DEX (10 µM, 1 µM, 0,1 µM, 0,01 µM) and analyzed for up to 60 h Analysis of leaves: Perforated or not-perforated leaves (age of plants not specified) of plants dipped in 10 µM DEX for up to 2 days Analysis of whole plants: Soil grown plants (age not specified) fumigated with 1 µM DEX overnight and analyzed after 5 days Soil grown plants (age not specified) sprayed with 1 µM DEX and analyzed after 4 days	Lee et al. 2025

Presents the publications identified by the authors. The intention was to include all relevant works; however, it is possible that some publications are not covered

Various developmental processes across diverse dicotyledonous plant species were studied with aid of the GR-LhG4/pOp system. These include 1) small herbs—edible weed from mustard family *Cardamine hirsuta* and symbiotic nitrogen fixing legume *Medicago truncatula*, 2) cultivated tropical crops—yielding melon-like fruits *Carica papaya L*. (papaya) and hybrid orange plants *Citrange carrizo* (*Citrus sinensis* × *C. trifoliata*), and 3) an angiosperm tree *Populus.* In these species various plant parts were studied. In *C. hirsuta,* GR-LHG4/pOp was used to analyze the role of *RCO (REDUCED COMPLEXITY)* in leaf shape determination. *RCO* induced expression, in *rco* mutant plants, enabled researchers to decipher which genes are transcribed due to the RCO presence (Hajheidari et al. 2019). Leaves (this time their abscission) were also studied in *Populus*. Induction of the Mg-dechelatase enzyme expression, which causes removal of Mg^2+^ from chlorophyll, led to yellowing of leaves, emission of ethylene, development of abscission layer and finally abscission of the leaf (Ito et al. 2022). In transgenic *M. truncatula* plants, researchers studied roots and used GR-LhG4/pOp for gene silencing by expressing YFP and YFP hairpin RNA under the control of the pOp promoter. Moreover, an endogenous *M. truncatula* gene, *ACETYL-CoA CARBOXYLASE* (*AccASE* in e.g. Liu and Yoder 2016*/ACCase* in e.g. Konishi et al. 1996; Szczepaniak et al. 2018) was silenced using ACCase hairpin RNA (Konishi et al. 1996; Liu and Yoder 2016; Szczepaniak et al. 2018). ACCase is crucial for fatty acid biosynthesis, and following DEX treatment, root growth was severely inhibited (Liu and Yoder 2016). RNAi and GR-LhG4/pOp systems were also used to silence endogenous genes of *C. papaya* L. to study fruit development. *C. papaya* L. plants were transformed with siRNA sequence targeting mRNA of enzymes 1-Aminocyclopropane-1-carboxylic Acid (ACC) oxidase 1 and 1-Aminocyclopropane-1-carboxylic Acid (ACC) oxidase 2 involved in ethylene synthesis and, consequently, fruit ripening. Fruits harvested from transgenic plants were sprayed with DEX which led to delayed ripening time (Sekeli et al. 2014). The GR-LhG4/pOp system also appears to be functional in other fruit-bearing species *Citrange carrizo*, as *GUS* reporter gene expression was successfully induced by watering soil-grown plants with DEX (Rossignol et al. 2014); however, no further functional validation was published in terms of this species. Non-fleshy fruits have also been studied with the GR-LhG4/pOp system (Eng et al. 2026). *C. hirsuta* seeds are characterized by explosive dispersal strategy dependent on distinctive SCW (secondary cell wall) pattern formation in *endb* cells, which distinguishes them from the non-explosive seeds of *Arabidopsi*s (Emonet and Hay 2024). Results obtained using GR-LhG4 suggested that cortical microtubules are required to properly pattern SCW of *endb* cells, which is essential for explosive valve coiling (Eng et al. 2026).

The GR-LhG4/pOp system was shown to be functional also in monocots. For *Oryza sativa* (rice) at first, the system had been codon optimized and tested with maize ubiquitin promoter driving LhGR expression and *GUS* reporter gene under pOp control. The expression was induced already in nanomolar DEX concentrations, and in concentrations 10 µM and higher severe growth defects were observed (Vlad et al. 2019). It was speculated that codon optimization causes much too efficient expression of LhGR, leading to non-specific binding and thus to growth defects. However, use of non-codon-optimized rice systems had no negative effects on plants even in DEX concentrations exceeding 10 µM (Samalova and Moore 2021). In rice so far only YFP has been utilized as an effector gene, nonetheless this study suggests that the GR-LhG4/pOp system can be employed not only in dicots but also in monocots; and that it works for rice seedlings and for mature soil-grown plants.

Lately, the GR-LhG4/pOp induction system was evaluated for its usefulness in the second monocot species *Setaria viridis* (a model species for C4 grasses) (Lee et al. 2025). The induced expression of anthocyanin pigmentation regulatory TFs *SvR1* and *SvC1* was sufficient to cause anthocyanin pigmentation in *S. viridis* mesophyll protoplasts and whole plants. However, this study also pinpointed limitations of the GR-LhG4/pOp system and the need for its further optimization in some monocot species. DEX was shown to have a limited diffusion and uptake in the leaves, and some minimal undesired alterations in plant morphology were observed. However, the authors also showed that a glucocorticoid other than DEX (i.e. triamcinolone acetonide) much more effectively penetrates leaf tissues. The induction was still not fully uniform, but inductor application procedures could potentially be improved for greater effectiveness. The authors noted that they also tested application of the inductor via watering plants, but detailed data is not provided. The observed undesired impact on *S. viridis* morphology could be caused by the use of rice codon-optimized GR-LhG4; and thus potentially could be limited by taking advantage of non-codon-optimized system, as was shown for rice (Lee et al. 2025).

### Integration of genome editing CRISPR/Cas technique and induction systems

CRISPR/Cas has transformed bioengineering and opened new perspectives for the precise editing of plant genomes for functional genetics studies and the generation of new crop varieties (e.g. Zhu et al. 2020; Rynjah et al. 2025). In most cases, strong, constitutive promoters have been used to drive Cas9 expression, which may lead to the accumulation of the Cas9–sgRNA complex in cells, and consequently, to undesired off-target effects (e.g. Zhang et al. 2018) and edits in essential genes may cause pleiotropic developmental defects, sterility or even lethality. Therefore, spatiotemporal control of the CRISPR/Cas9 system has been implemented to overcome the problems.

In *Arabidopsis,* the CRISPR/Cas9 technology has been integrated with the GR-LhG4/pOp system (CRISPR-KILL technology described above; Gehrke et al. 2023), with the β-estradiol-inducible XVE system (Wang et al. 2020; Donà et al. 2023; Lu et al. 2025) and heat shock systems (Liang et al. 2023). In Wang et al. (2020) efficient generation of target gene knockouts (particularly (*PLETHORA1* (*PLT1*) and *PLT2*) at desired times and cell types only (four specific, XVE-based inducible promoters) was achieved in *Arabidopsis* roots (Wang et al. 2020). In Donà et al. (2023) and Lu et al. (2025) the integration of the XVE induction system with CRISPR/Cas enabled the generation of inducible toolsets for tracing the division history of cells during root development (through fluorescent protein activation in Donà et al. 2023) and during whole-plant regeneration from root explants (through DNA barcoding in Lu et al. 2025). The heat shock inducible approach produced clear phenotypic outcomes when mutagenesis was directed at the *BRI1* and *PDS* genes independently at two developmental growth stages (seedling and adult flowering plants) (Liang et al. 2023). The heat shock approach was also used to trigger Cas9 expression in rice (to disrupt the *GUS* marker gene and the *PDS* gene; Nandy et al. 2019)) and in maize (for targeted gene insertion at specific time points between plant material transformation and mutant selection; Barone et al. 2020). The β-estradiol based systems were additionally used to disrupt the *PDS* genes in tobacco (Ren et al. 2020) and rice (Tang et al. 2016) and to provide a chemically controlled upregulation of target genes (CRISPR-a system) (Zhang et al. 2024). The latter approach, i.e. CRISPR-a, was used to accelerate plant regeneration from callus cultures of dicot plants *Medicago sativa* (alfalfa; vital agronomic legume plant) and *Fragaria vesca* (woodland strawberry; model diploid progenitor species for non-climacteric fruits) and monocot plant *Leymus chinensis* (*s*heepgrass; forage crop and dominant perennial grass in Eurasia) by inducible expression of various morphogenic genes (Zhang et al. 2024). A special inducible approach was the development of a Virus-inducible CRISPR/Cas9 system engineered to confer resistance to beet severe curly top virus (BSCTV) in *Arabidopsis*. By incorporating the BSCTV-inducible promoters, the CRISPR/Cas9 vectors are rapidly trans-activated upon infection with BSCTV, which efficiently inhibits BSCTV accumulation (Ji et al. 2018).

Notably, as it is not the main scope of this review article, in this short chapter we did not cover all approaches of inducible genome editing in plants (e.g. those related to the Cre/lox system). It is noteworthy that various techniques can nowadays be combined in a single transgenic plant. An example is a study performed in *Marchantia polymorpha,* in which the function of essential genes was analyzed by means of simultaneous mutagenesis of endogenous target gene(s) by the CRISPR/Cas9 system and of a conditionally removable complementation gene introduced as a transgene. The latter achieved by the presence of Cre recombinase fused to the rat glucocorticoid receptor domain (GR) under the control of the heat shock promoter (Sugano et al. 2018).

## Perspectives

Combining CRISPR/Cas9 technology with inducible systems allows the precise manipulation (knockout or activation) of target genomic site(s) on demand (in desired cell types and at desired times) and, in consequence, helps to limit off-target effects and to effectively adapt the system to experimental questions. Traditional uses of various induction systems have fundamentally accelerated functional genomic studies; therefore, integrating these systems with revolutionary CRISPR technologies will unprecedentedly further accelerate basic research in model plants and in economically valuable species. GM crops based on CRISPR/Cas9 systems can provide means to develop desirable new crop varieties with increased resistance to abiotic and biotic stresses and to boost crop yields without expanding farmland. Off-target effects have raised strong regulatory concerns, but inducible approaches provide a strategy to overcome this drawback. The potential disturbance of the gene pool in GM crops (by the introduction of exogenous genes from organisms such as bacteria or unrelated plants) also raises considerable safety concerns. Notably, an inducible CRISPR/Cas9-based system may provide a potential solution to this problem as well. In *Arabidopsis*, undesirable genetic transgenes could be “cleaned” from the final plant product by integrating additional heat shock inducible components (Hu and Yu 2022).

## Conclusions

Collectively, the cited studies indicate that the GR-LhG4/pOp system is a versatile method for the inducible expression of various types of transgenes in both dicotyledonous and monocotyledonous plant species. A wide range of promoters can be used, and inductors can be applied at different stages of development in a precisely adjusted way. All types of organs/structures/tissues can be analyzed as defined by researchers timepoints after induction and, thus, experiments aiming to better understand a plethora of developmental processes can be performed. This sophisticated system allows precise spatiotemporal synchronization of transgene expression, and thus, circumvents the problem of unspecific changes accumulating with time (and age of plants).

Moreover, the body of evidence presented in the cited articles demonstrates that DEX is an efficient and apparently non-toxic inductor. Therefore, the GR-LhG4/pOp system allows analyzing both the immediate effects of transgene expression (due to rapid activation after DEX application) and the long-term effects of its presence within a given tissue type. Importantly, GR-LhG4 should not be codon optimized for monocotyledons. Moreover, GR-LhG4/pOp has been shown to be inactive in the absence of DEX and it does not cause non-specific growth defects. However, certain inductor application methods can make the results less accurate (e.g. spraying vs local paintbrush application) and DEX application procedure must be adjusted to the plant structure under study. Limited knowledge of DEX mobility within the plant body can make it quite demanding. Although finding a cell/growth stage specific promoter can still also be challenging, the use of the GR-LhG4 system is expanding. In consequence, the availability and the number of driver lines increase, and thus tissue-specificity in transgenes expression diversity is also expected to grow in number. Likewise, our knowledge of various DEX application procedures is expanding, and new methodologies are being developed. Therefore, a suitable experimental design for DEX administration can be established for a given research question.

The GR-LhG4/pOp system is widely used in *Arabidopsis* research. Currently, there is a growing trend to translate the knowledge obtained in *Arabidopsis* to other plant species, which can help us better understand the world surrounding us and develop plants with improved performance to boost yield, stress resistance, and nutrient uptake. Moreover, certain developmental aspects just need to be studied in species other than *Arabidopsis*. For example, *Arabidopsis* does not produce fleshy fruits, as many agriculturally significant plants do, and it does not take advantage of the mycorrhiza (a process that enables a better absorption of water and nutrients from the soil). Therefore, *Arabidopsis* lacks various specific regulatory and metabolic pathways. Luckily, the GR-LhG4/pOp system was shown to be a universal tool, functional in economically significant species: dicots, such as papaya and *Populus*, and monocots, such as rice. Therefore, most likely it can be applied to a wide range of species, and the only limitation can be whether a particular species/cultivar can be made transgenic. Research on agriculturally significant plants rises, and accessibility of advanced, low-cost molecular tools has the potential to boost our understanding of this research field as well. The use of the GR-LhG4/pOp system in economically valuable species is still limited. Notably, to our knowledge, the system GR has so far been integrated with the CRISPR/Cas9 technology only in *Arabidopsis* (Gehrke et al. 2023). The employment of this universal, versatile, effective and non-toxic method for induced transgenes expression has, however, potential to expand.

Climate change is causing normally occurring weather phenomena (e.g. droughts, heat waves, freezing temperatures) to become more severe and more frequent. There is, therefore, a growing need to better understand plant-environment interactions, for example to develop plants better adapted to a new reality of their cultivation sites (also those agriculturally relevant). The GR-LhG4/pOp system has already shown to be useful for analyzing plant stress response and tolerance. For example, it has been used to study *EXPA1* and drought stress (Balkova et al. 2025) or *ARR22* and freezing stress tolerance (Kang et al. 2013). The climatic changes that we encounter proceed relatively fast; thus all tools that will allow researchers to study environmental impact on plants’ growth and development in a faster and more precise manner are of value. *Arabidopsis* still appears to be a great model plant for basic research, as various resources have already been established. Knowledge can thus be more easily obtained and methods established with the use of *Arabidopsis* can then be used in application studies in other plant species. It is, therefore, highly plausible that the use of the GR-LhG4/pOp system in research on environmental stresses (also that integrating the CRISPR/Cas technique) will continue to expand.

## Data Availability

Data sharing not applicable to this article as no datasets were generated during the current study.
